# A Proposed Regulatory Review Model to Support the South African Health Products Regulatory Authority to Become a More Efficient and Effective Agency

**DOI:** 10.34172/ijhpm.2020.213

**Published:** 2020-11-23

**Authors:** Andrea Keyter, Sam Salek, Shabir Banoo, Stuart Walker

**Affiliations:** ^1^Department of Clinical and Pharmaceutical Sciences, School of Life and Medical Sciences, University of Hertfordshire, Hatfield, UK.; ^2^South African Health Products Regulatory Authority, Pretoria, South Africa.; ^3^Faculty of Health Sciences, University of the Witwatersrand, Witwatersrand, South Africa.; ^4^Centre for Innovation in Regulatory Science, London, UK.

**Keywords:** South Africa, Review Model, ZAPAR, SAHPRA, Global Benchmarking Tool

## Abstract

**Background: **National regulatory agencies of various sizes and maturity levels, including the South African Health Products Regulatory Authority (SAHPRA), have had to revise systems and re-engineer processes in order to adapt to the new regulatory environment and increase the effectiveness of regulatory operations. This study aimed to develop a new regulatory review model for improved regulatory performance, underpinned by the parameters of the World Health Organization Global Benchmarking Tool (WHO GBT) that support strengthening of regulatory systems.

**Methods:** A new enhanced model for regulatory review, was developed based on the key recommendations from 6 studies, previously conducted by the authors, that were identified as fundamental elements in enhancing regulatory performance. The elements selected to define the new regulatory review model were endorsed through the integration of the parameters of the WHO GBT that, when embedded within regulatory systems, support enhanced regulatory performance.

**Results:** Opportunities for improvement in regulatory performance were identified and include quality measures; monitoring and evaluating review times; a risk-based evaluation; transparency and communication; and training and education. An improved model for the South African regulatory review and benefit-risk (BR) assessment supported by quality decision-making was proposed as well as recommendations for the application of risk-stratification strategies, strengthening of reliance networks, reinforcing good regulatory practices (GRPs) and enhancing transparency.

**Conclusion: **If implemented the proposed improved regulatory model may pave the way towards more efficient and transparent, streamlined review processes, coupled with increased consistency, evidence-based decision-making practices, reduced timelines and improved patients’ access to new medicines in South Africa.

## Background

**Key Messages**

**Implications for policy makers**

- The studies described here have resulted in recommendations for an improved model for the regulatory review of medicines by the South African Health Products Regulatory Authority (SAHPRA) and provided a baseline against which future improvements implemented by SAHPRA may be measured.
- Following the implementation of the SAHPRA re-engineered processes it would be useful to compare the new registration process and regulatory review model of SAHPRA against other similar-sized national regulatory agencies.
- Provided that the recommendation to identify and routinely monitor and evaluate the milestones in the regulatory review process is implemented, it would be useful to analyse the timelines achieved between these milestones in order to accurately determine the time taken by SAHPRA to review an application and the time taken by the applicant to provide the required response/s to SAHPRA.
- Considering the intention of SAHPRA to implement facilitated regulatory pathways (FRPs), it would be valuable to study the overall median approval timelines achieved for full, abridged and verification reviews and their impact on patients’ access to medicines.
- The use of a structured universal template for benefit-risk (BR) assessment both for SAHPRA and for regional initiatives has been encouraged. This would support predictable, transparent and quality decision-making and provide an effective approach for communicating BR decisions made through the use of collaborative initiatives and could form the basis of a public assessment report (PAR).

** Implications for the public**

The increasing volume of applications received by the former national regulatory agency in South Africa, coupled with resource constraints, outdated manual document management systems and legislative constraints, resulted in the development of a significant backlog in medicine registration and an unprecedented extension of review timelines, which were much longer than those achieved by regulatory authorities in developed and comparable emerging economies. Undoubtedly, the delayed approval times for medicines in South Africa negatively impacted patients’ access to vital medicines. The new national regulatory agency in South Africa, the South African Health Products Regulatory Authority (SAHPRA) has been working to increase its resources and improve its processes. It is hoped that this proposed improved review model will be considered by SAHPRA and will pave the way towards efficient and transparent, streamlined review processes, coupled with increased consistency, evidence-based decision-making practices, reduced timelines and improved patients’ access to new medicines.

The effective regulation of medicines, the strengthening of regulatory systems and the improvement of regulatory performance have become the focus for national regulatory authorities (NRAs) and governments worldwide. The NRAs are responsible for protecting and promoting public health, implementing rigorous regulatory standards and maintaining an assured supply of medicines which are safe, effective and of good quality.^1-3^ However, global mounting pressure on NRAs of all sizes and capacities have been noted due to the larger volumes of marketing authorisation applications received, the complexity of the submissions and the increased categories of medicines.^4^ Whilst patient-focused, evidence-based, risk-oriented, transparent, effective and flexible practices are the mainstay of medicines regulation,^5^for many NRAs, particularly in emerging economies with resource-limited settings, achieving these types of practices has not been a reality.^6^ In response to these challenges, NRAs of various sizes and maturity levels have had to revise legacy systems and re-engineer processes in order to adapt to the new regulatory environment and increase the effectiveness of regulatory operations.

###  Regulatory Challenges in South Africa

 The Medicines Control Council (MCC), the past NRA in South Africa, had historically faced similar difficulties. The increasing volume of applications received by the MCC, coupled with resource constraints, resulted in the development of a significant backlog in medicine registration and an unprecedented extension of their respective review timelines.^7,8^ The approval timelines for new active substances (NASs) in South Africa were much longer than those achieved by NRAs in developed and comparable emerging economies.^9^ The MCC regulatory review process was deemed to be inherently slow as a result of insufficient human and financial resources, outdated manual document management systems and legislative constraints that did not support the use of facilitated regulatory pathways (FRPs).^7,10^ Undoubtedly, the delayed approval times for NASs in South Africa negatively impacted patients’ access to medicines.

###  Harmonisation, Reliance and Recognition

 Efforts to address the challenges faced by NRAs in low- and middle-income countries have focused on strategies for identifying and performing core regulatory functions that have to be undertaken directly by NRAs to meet country or regional needs.^6,11^ The NRAs have also been encouraged by the World Health Organization (WHO) to consider regulatory convergence and to collaborate with and recognise the work done by other NRAs in order to avoid the duplication of regulatory efforts and to ease the regulatory burden.^6,11,12^At the core of harmonised regulatory activities lies the need to reach convergence in regulatory requirements. In order to do so, NRAs are required to function at the necessary maturity level. Through harmonisation initiatives, technical requirements for safety, quality and efficacy may be standardised, the regulatory burden faced by many agencies may be reduced and the duplication of regulatory efforts may be avoided.^11^ The use of FRPs may also be considered as a mechanism to expedite regulatory decision-making in the review of applications for the registration of NASs.

 Technical support, underpinned by efforts promoting regulatory convergence, has been provided by WHO to Member States. The WHO has initiated collaborative activities between various countries and regions and through these harmonisation initiatives participating NRAs have been able to exchange consolidated information without challenging the sovereignty of the participants.^5^ Global trends for convergence and reliance have been taken into account in the African region as reflected through the informal consultations initiated at the International Conference of Drug Regulatory Authorities, held in Bern, Switzerland, in September 2008. As a result of these discussions a WHO concept paper was developed to institute the African Medicines Registration Harmonization Initiative to support the harmonisation of medicine registration within and across Africa.^5^It is further anticipated that the establishment of African Medicines Agency may further support the regulatory systems of NRAs and build regulatory capacity within countries in the African region.^2^

###  WHO Global Benchmarking Tool

 International benchmarking against mature NRAs has driven many agencies to strive towards the implementation of pragmatic solutions to address regulatory inefficiencies. The WHO has developed a global benchmarking tool (GBT) that has been used to perform an evidence-based assessment and comparison of NRAs. The WHO GBT is used by the WHO to assess the regulatory systems of NRAs in Member States, as mandated by the World Health Assembly Resolution 67.20 on regulatory system strengthening for medical products.^13,14^ The benchmarking methodology embedded within the WHO GBT enables the WHO to identify both strengths and areas for improvement within the agencies’ regulatory system. The GBT is used to evaluate each of the nine component regulatory functions of the regulatory system against a series of sub-indicators. These functions include: national regulatory systems; registration and marketing authorisation; vigilance; market surveillance and control; licensing establishments; regulatory inspection; laboratory testing; clinical trial oversight and lot release. Fact sheets have been developed to describe the scope and requirements for each sub-indicator. During the assessment, NRAs are required to provide evidence supporting the implementation of each of the sub-indicators.

 A number of the WHO GBT sub-indicators highlight the importance of formalising the implementation of a quality management system (QMS) and good review practices (GRevPs). The sub-indicators require NRAs to demonstrate the effective application of quality decision-making practices (QDMPs) in regulatory decision-making and support the publication of regulatory decisions in the public domain. The sub-indicators endorse the monitoring and evaluating of regulatory performance, making use of effective electronic document management systems (EDMSs) and participation in regional and/or global networks to promote harmonisation and collaboration. Each sub-indicator is linked to a ‘maturity level’ rating. The measure of maturity level is based on the concept adapted from the International Standardization Organization (ISO 9004 standard) that provides guidance on quality management and the quality of an organisation to achieve sustained success.^14^ The GBT facilitates an assessment of the maturity level of an NRA on a scale of 1 (existence of some elements of regulatory system) to 4 (operating at advanced level of performance and continuous improvement). The NRAs that are operating at a maturity level of 3 and above are considered to be competent in effecting regulatory mandates and are listed by the WHO as such. The application of the WHO GBT in the assessment of NRAs in WHO Member States provides an opportunity for those that are operating at lower maturity levels or those in resource-limited settings to rely on or recognise the regulatory decisions of WHO-listed NRAs.

###  Changing the South African Regulatory Environment

 The drive for the establishment of a more effective regulatory framework in South Africa has been evident for the past 2 decades. In June 2017, the Medicine and Related Substances Act, 1965 (Act 101 of 1965), was amended to allow for the transition of the MCC to the South African Health Products Regulatory Authority (SAHPRA). Promising regulatory reform, this new era provided an opportunity to study the past practices of the South African NRA, with a view to enhancing regulatory operations and the responsiveness of the NRA to the advancing new regulatory landscape. Similar to other NRAs, SAHPRA is working toward the development and improvement of its regulatory capacity. At a workshop convened by the Centre for Innovation in Regulatory Science (CIRS), on the risk-based evaluation of medicines,^15^ several NRAs expressed an interest in applying risk-based evaluation approaches that focused on reliance on the work of other trusted NRAs, and SAHPRA is also exploring the practical implementation of such models.

 The need for agencies to consistently measure their performance against established target times, an important GBT parameter, can be facilitated through the CIRS Optimising Efficiencies in Regulatory Agencies (OpERA) tool.^16^ The OpERA tool was developed through the identification of common milestones in the regulatory review process by regulatory agencies and regional initiatives so that participating agencies could identify where time is spent in their processes, delineate performance goals and transparently monitor progress toward those goals.^16^

 As SAHPRA moves forward with its objective of regulatory reform to improve median approval times and patients’ access to medicines, it is important that the agency has the relevant capabilities and decision-making frameworks in place to ensure the efficient application of resources. Because of the interest of stakeholders in registering new medicines in South Africa and the increasing backlog in registration, there was a need for a comprehensive study to support the regulatory environment in the region underpinned by a new regulatory review model. The development of such model will be informed by an evaluation of the outcomes and recommendations derived from 6 studies, previously conducted by the authors. The model, synthesised as a result of this study, will be underpinned by the same parameters that form the basis of the GBT, used by the WHO to perform an evidence-based assessment of the performance of regulatory systems of NRAs.

 Therefore, the aim of this study was to develop a regulatory review model for enhanced regulatory performance.

## Methods

 In order to develop a new enhanced model for regulatory review, it was necessary to examine the current model implemented by the South African NRA. The model of review used by the NRA was identified (study 1), the overall timelines achieved using this model were described (study 2 and study 3) and the regulatory performance achieved, using this model, was compared to that of similar NRAs (study 4). The outcomes of study 5 described whether the model used by the NRA was justified or required improvement and the transparency of the outcome of the regulatory review was evaluated (study 6). The recommendations from these 6 studies, previously conducted by the authors, were analysed in order to extract the key recommendations that are fundamental in informing the design of the enhanced model for regulatory review.

 A questionnaire technique was used in study 1 to identify the models of review that are being used within the authority, identify target times and the main activities between milestones for registration, and identify the organisational structure, and the capacity of the authority. The questionnaire was completed with a view to analysing the quality measures that are currently in place, identify areas of capacity constraints, and to provide a baseline for the current review process, considering the transition to the newly established SAHPRA. This was followed by collecting data in study 2 and 3 to reflect the overall approval times for NASs registered by the South African NRA during the period 2015–2018.

 To arrive at a plausible conclusion with respect to the collected data described above and fulfil the study objectives, it was necessary to contextualise the South African regulatory environment and how it compares to other similar countries around the globe. Consequently, in study 4 the data were compared with that of 4 other countries (Therapeutic Goods Administration, TGA, of Australia; Health Canada; the Health Sciences Authority of Singapore; Swissmedic) chosen on the basis of the size of the agencies and the patient population they served, the year since established and the nature of the review model (full assessment) applied.

 To test the transparency of the outcome of the regulatory review comparing South Africa with 4 other regulatory authorities. The public assessment reports (PARs) of ertugliflozin L-pyroglutamic acid, erenumab and durvalumab recently published by the regulatory bodies in Australia, Europe, Canada and the United States were compared with the validated Universal Methodology for Benefit-Risk Assessment (UMBRA) benefit-risk (BR) template in study 5 to determine whether the BR decision had been documented in a systematic and structured manner. The approach initiated by the SAHPRA to document and communicate BR decisions was also evaluated in study 5.

 The next step was to examine if there was plausible justification for the review model (full assessment) applied by the MCC or the successor authority, SAHPRA. To this effect, a 5-part questionnaire, the Abridged Review Process Profile^9^ was used in study 6 to identify the criteria and current practices that were applied by NRAs for implementing an abridged review process.

 The elements selected to define the new regulatory review model were endorsed through the integration of the parameters of the WHO GBT that, when embedded within regulatory systems, support enhanced regulatory performance.

###  Data Processing and Analysis

 The Excel syntax was used to manage and analyse the data collected for this exploratory study during the period 2015–2018. Furthermore, the characteristics of the medicinal products submitted to the authority for registration were described. The review type (fast track/standard) applied to each regulatory submission was identified as well as the origin (multinational company/local company) of the submission and the definition of the milestones within the review process. Descriptive statistics such as summary scores, frequencies, percentages, etc were applied. The median timelines for each of the milestones within the review process as well as the median overall approval times were calculated and analysed. Median approval times by product type and therapeutic area were determined and all data were analysed as calendar days. In addition, the MCC and SAHPRA regulatory processes and frameworks conducted during 2018-2019 were evaluated against the validated WHO GBT sub-indicators and global efforts toward regulatory convergence and collaboration^5^ to develop recommendations for an improved regulatory model for SAHPRA, including the use of the OpERA tool to monitor and evaluate milestones and overall timelines.^16^

## Results

 A summary of the recommendations of the 6 studies, that were previously conducted by the authors, have been identified to be pivotal in the development of a regulatory review model for enhanced regulatory performance (Table). This demonstrates how each recommendation translates into each element of the evidence-based model that has been created. In turn, these elements are reinforced by the parameters of the GBT that are implemented in efficient and effective regulatory systems.

**Table:** Summary of the Methodologies and Recommendations Informing the Development of the New Regulatory Model Based on the Principles of the WHO GBT

**Study Number**	**Aim**	**Method**	**Key Recommendations From the Study**	**Corresponding Elements Within the New Regulatory Model (Figure 2)**	**Corresponding GBT Parameters Endorsing the Elements of the Model**
Study 1^7^	Examine the regulatory review process applied by the MCC	A questionnaire was completed by the MCC to describe the organisation of the authority, record key milestones and timelines in the review process and to identify GRevPs	Apply a risk-based approach to the review of NASs using FRP Formalise the implementation of the QMS Define timelines and measure milestones in review process and overall approval time	The following 5 areas for improvement were identified to be common amongst the recommendations from the 6 studies conducted. These 5 elements encompass all the recommendations from each study and were deemed to be critical in informing the development of the new regulatory model	The GBT is used to evaluate each of the nine component regulatory functions of the regulatory system against a series of sub-indicators For the purpose of this study reference was made specifically to the sub-indicators of the regulatory functions of the national regulatory systems and marketing authorisation
Study 2^10^	Provide the historical context supporting the new regulatory environment in South Africa and the transition from the MCC to SAHPRA	A review was conducted of the history of the enabling legislation supporting the establishment of SAHPRA and the similarities and differences between the MCC and SAHPRA were compared	Training and skills development of regulatory expert reviewers Establish committee structures within the NRA for ad hoc consultation Monitoring and evaluating Formalise the QMS Apply a risk-based approach to the review of NAS using FRP	**Quality Measures** Establish a dedicated quality management unit Formally implement QMS, GRevPs and GRelPs Codify and institutionalise the quality policy, SOPs, guidelines and assessment templates Use the UMBRA BR Summary Template as the guide for BR assessment and the outline for the preparation of the ZAPAR Employ ODMPs	RS05.01: Top management intervention is required to demonstrate commitment and leadership to develop and implement a QMS
					RS05.02: The quality policy, objectives, scope and action plans for the establishment of the QMS must be in place and be communicated to all levels
					RS05.04: Enough competent staff must be assigned to develop, implement and maintain the QMS
					RS03.05: The NRA is required to promote GRPs
					MA04.10: The formal implementation of GRevPs is required
Study 3^8^	Evaluate the timelines of the milestones of the South African review process and the overall approval process for NASs	Data identifying the milestones and overall approval times for NASs registered by the South African Agency during 2015–2018 were collected and analysed	Define timelines and measure milestones in review process and overall approval time Formally implement GRevP Apply the UMBRA Implement FRPs Apply regulatory trade-offs: use surrogate endpoints for expedited market authorisation Develop and implement ICT system Formalise the QMS	**Monitoring and Evaluating** Identify the milestones in the regulatory review process Formalise the target timelines for the review process Record and measure the timelines for each of the milestones Monitor the timelines to ensure that target timelines are met Embed the target timelines in performance contracts Prioritise the implementation of the EDMS to ensure the accurate tracking of applications and recording of the timelines achieved	MA04.06: The establishment of timelines for the assessment of applications and an internal tracking system are required to follow the targeted timeframes
					MA06: The use of a mechanism to monitor regulatory performance and output is required
					MA06.02: The establishment and implementation of performance indicators for registration and/or market authorisation activities is required
					RS10.01: The monitoring, supervision and review of the performance of the NRA is required using key performance indicators
Study 4^17^	Compare the registration process and the regulatory review model of the MCC to that of 4 other similar-sized regulatory authorities	A questionnaire was used to describe the structure, the registration process, good review and decision-making practices of the MCC Similar questionnaires were also completed and validated by Australia’s TGA, Canada’s Health Canada, Singapore’s HSA and Switzerland’s Swissmedic	Define timelines and measure milestones in review process and overall approval time Formally implementing GRevP Apply UMBRA Implement FRPs and apply a risk-based approach to regulatory review process Establish committee structures within the NRA for ad hoc consultation Enhance transparency and communication through development and publication of public assessment report (ZAPAR)	**Apply a Risk-Based Approach to Review** Formalise FRPs in order to conserve limited resources, avoid duplication of regulatory effort and shorten timelines for medicine registration Consider alternatives to the full review process, such as the abridged review and verification review Rely on or recognise reference agencies’ assessment reports Rely on or recognise the regulatory decisions of reference agencies Strengthen collaborations and initiatives for joint reviews/work-sharing	RS03.04: Reliance on the decisions of other mature NRAs through documented policy, procedures and/or mechanisms must be formalised
					RS09.01: NRAs are encouraged to participate in a regional and/or global network in order to promote convergence and harmonisation efforts
Study 5^18^	Review the PARs available in the public domain against the UMBRA BR Template using a case study approach Evaluate the approach initiated by SAHPRA to document and communicate the BR decision	PARs for 3 NASs published by NRAs in Australia, Europe, Canada, and the United States were compared with the validated UMBRA BR Template to evaluate the BR decision documentation A focus group discussed the use of PARs as potential knowledge management tools for stakeholder understanding of regulatory decision-making The SAHPRA approach to document and communicate the BR decisions was evaluated	Perform BR assessment in a structured, systematic documented manner Preparation and publication of a ZAPAR to communicate the BR decision Use UMBRA BR Template for BR assessment and as an outline for the public assessment report (ZAPAR)	**Transparency and Communication** Enhance stakeholder relationships through improved communication and transparency Publish updated lists of SAHPRA licence holders and medicine registrations Facilitate online submission and tracking of applications Publish SAHPRA’s summary basis of decision in the form of the public assessment report (ZAPAR)	MA05: NRAs must ensure that mechanisms exist to promote transparency, accountability and communication
					MA05.01: NRAs are required to ensure the availability of a website or other official publication that is regularly updated
					MA05.02: NRAs are required to publish an updated list of all medicines granted market authorisation
					RS09.04: NRAs are required to publish information on marketed medical products, authorised companies and licensed facilities
					MA05.03: NRAs are required to publish the summary technical evaluation reports for approved applications of marketing authorisation in the public domain
					RS09.03: NRAs are required to publish the NRA decisions related to regulatory activities in the public domain
Study6^19^	Identify criteria and current practices for implementing an abridged review process and understanding barriers and enablers in utilising reliance models	A questionnaire was completed by 6 NRAs to determine criteria and current practices for implementing an abridged review process Two focus group discussions were conducted on the practical implementation of an abridged review process based on GRelP	Formalising the implementation of GRelP; Place reliance on trusted NRAs Verify sameness of NAS applications submitted to SAHPRA Limit the scope of the abridged review to a: Detailed review of clinical data Review of the quality data and non-clinical data only in the event of query; and Selective review of human pharmacology data	**Training and Education** Training programs should be formalised Priority should be placed on the professional development of internal and external assessors Ongoing skills development may be maintained through the initiation of mentorship programmes The development of additional capacity will contribute towards enhanced regulatory performance and shortened timelines for regulatory review	MA03.01: Sufficient competent staff (education training skills and experience) should be assigned to perform marketing authorisation
					MA03.03: The development, implementation and annual updating of the training plan is required
					MA03.04: Performing and maintaining records of staff training activities is required
					RS05.14: The establishment of a mechanism to evaluate and demonstrate the effectiveness of training activities is required

Abbreviations: WHO GBT, World Health Organization Global Benchmarking Tool; BR, Benefit-Risk; FRPs, facilitated regulatory pathways; GRPs, good regulatory practices; GRelPs, good reliance practices; GRevPs, good review practices; SOPs, standard operating procedures; HSA, health science authority; ICT, information and communications technology; MCC, Medicines Control Council; NASs, new active substances; NRA, national regulatory authority; PARs, public assessment reports; QMS, quality management system; SAHPRA, South African Health Product Regulatory Authority; TGA, therapeutic goods administration; UMBRA, Universal Model for Benefit Risk Assessment; ZAPAR, South African Public Assessment Report; ODMPs, quality decision-making practices.

*Studies 1 and 2: *The evaluation of the status of the MCC, prior to the establishment of SAHPRA in terms of its organisational structure and the regulatory review process for NASs was the focus of the 2 studies and included an assessment of the level of implementation of good regulatory practices (GRPs) and GRevPs by the MCC and provided further historical context supporting the new regulatory environment in South Africa and the transition from the MCC to SAHPRA.^7,10^ The results of these studies documented the regulatory approval time and the associated milestones within the MCC review process for NASs from 2015-2017, illustrating that the MCC in its capacity at the time was not able to achieve the target timelines for the regulatory review of NASs. Recommendations were made to support the implementation of a risk-based regulatory review process and the formalisation of reliance on the regulatory efforts of reference NRAs.

*Study 3: *This study reviewed the key milestones and metrics in the regulatory review process applied by the MCC for NASs from 2015-2018, including new chemical entities, biologicals and major line extensions and those embedded within the transitional process applied by SAHPRA for NASs registered during 2018.^8^In this study, the authors determined overall median approval time for NASs, reviewed the challenges and opportunities for expediting these timelines, and made recommendations for an improved regulatory performance in South Africa.

*Study 4:* The medicine review process applied by the MCC was compared with the processes applied by the agencies in Australia, Canada, Singapore and Switzerland. The comparison indicated that the timelines for the MCC medicine review process were considerably longer than those achieved by the comparative agencies. Recommendations made as a result of this study echoed the need for the formalised implementation of GRevP, routine metrics collection and a template for BR assessment to support consistent, predictable, transparent and timely regulatory review.^17^

*Study 5:* The assessment of the use of a BR framework in South Africa has also been explored. PARs from regulatory agencies in Australia, the Europe, Canada and the United States were compared with the validated UMBRA BR Summary Template to determine whether the BR decisions of those agencies had been documented in a systematic and structured manner. A focus group was also conducted to discuss the use of PARs and participants agreed that a standardised PAR template would support improved transparency and stakeholder understanding of regulatory decision-making. The approach initiated by SAHPRA to document and communicate BR decisions was evaluated and key recommendations for SAHPRA for the implementation of an effective approach for communicating BR decisions were developed. These include consideration of the UMBRA BR Summary Template as guidance for BR assessment as well as the use of this approach as an outline for the preparation of a proposed South African public assessment report (ZAPAR). The publication of the ZAPAR would promote the transparency of SAHPRA decision-making. It is also recommended that documented BR assessments, such as the PARs, may be relied on by other agencies in order to facilitate expedited review times.^18^

*Study 6:* A questionnaire was completed by regulatory authorities in Australia, Brazil, Canada, the Gulf Health Council, Israel, and Thailand to determine criteria and current practices for implementing an abridged review process. In addition, 2 focus group discussions were conducted on the practical implementation of an abridged review process based on “good reliance practices (GRelPs).” The results of this research facilitated the publication of recommendations for the implementation of an abridged review process in South Africa based on GRelPs.^19^

###  Improved New Proposed Model

**Figure 1:**
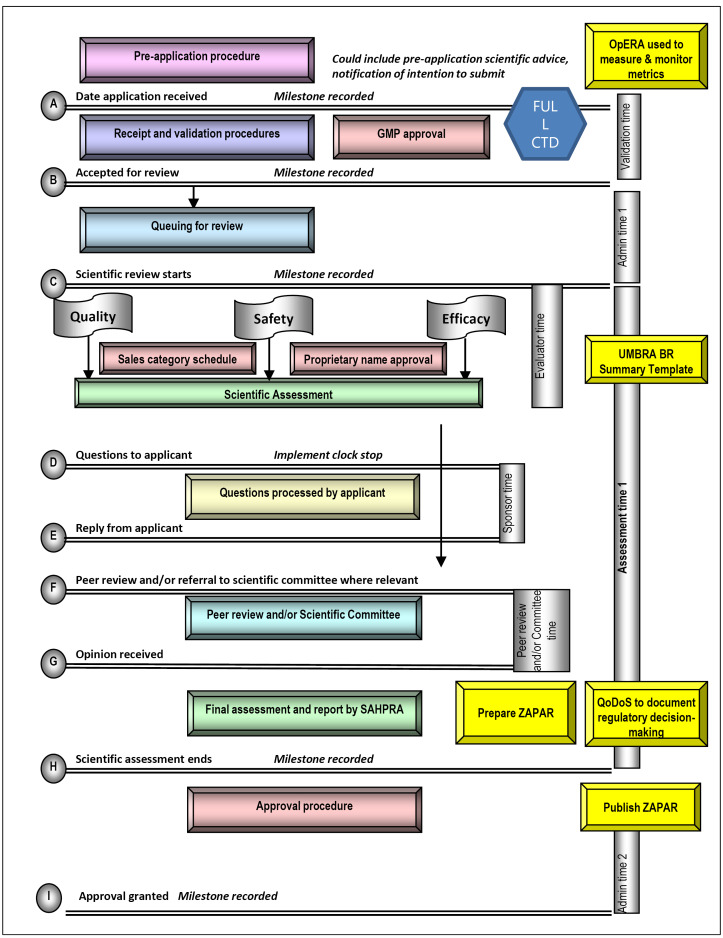
Proposed Model for the Improved Review Process. Adapted from Keyter et al^7^ and Keyter et al.^17^Abbreviations: BR, benefit-risk; CTD, common technical document; GMP, good manufacturing practice; OpERA, optimising efficiencies in regulatory agencies; SAHPRA, South African Health Products Regulatory Authority; QoDoS, quality of decision-making orientation scheme; UMBRA,universal methodologies for benefit-risk assessment; ZAPAR, South African Public Assessment Report.

 The proposed model for an improved full review process is illustrated in Figure 1. To be able to monitor and evaluate milestones and overall timelines it is necessary to implement an electronic tracking system such as that used in the OpERA tool.^16^ On receipt, the application will be validated and the good manufacturing practice (GMP) status of the manufacturing facility and laboratory will be verified. The application should not progress without confirmation of a positive GMP status for the relevant facilities listed in the application. A full Common Technical Document (CTD) should be submitted and full review of the quality/chemistry manufacturing and controls, safety and efficacy is highly recommended to be performed in parallel. The naming and scheduling of the NAS should also take place during this time. It would be of paramount importance that the applicants be given specified time to respond to any questions posed by SAHPRA and the time for evaluation of the response to such questions should be limited. Only one cycle of questions and answers should routinely be permitted with an additional cycle used only in exceptional circumstances. The UMBRA BR Summary Template is recommended to be used to conduct the evaluation of the clinical data and record the BR decisions. It is essential that assessment reports, prepared by SAHPRA during the review process, be peer-reviewed by the scientific committee. More frequent ad hoc consultation of a scientific expert committee should be limited to applications for market authorisation requiring expert review and recommendation.^17^ At this stage SAHPRA should consider the preparation of a PAR (ZAPAR) in order to document their regulatory decision and publish it in the public domain in order to enhance transparency. In addition, QDMPs should be evaluated using the Quality Decision Orientation Scheme (QoDoS).^20,21^

 The proposed model for a review process based on reliance should also be considered. The WHO has published a draft working document on good reliance practices in regulatory decision-making that describes the high-level principles and recommendations for the implementation of reliance practices.^22^ Once finalised; these principles should be integrated into the proposed reliance model. Such a review based on reliance could be performed for NASs that have been previously assessed and registered by one or more reference agencies recognised by SAHPRA,^19^ depending on whether it is an abridged, verification or recognition review. Only applications that are identical to those submitted to and approved by the reference agencies would be eligible for such a review. Specifications of the NAS including dosage form, strength, ingredients, indications, dose, warnings and precautions have to be identical to that of the NAS submitted to the reference agency. A closely similar product label would be acceptable. On submission, the applicant would be required to supply the full CTD, evidence of registration of the NAS by the reference agency, the list of questions to the applicant and the accompanying responses as well as any documented post-marketing commitments agreed prior to registration. It would be useful if the unredacted assessment report prepared by the reference agency to document the rationale for the reference agency’s regulatory decisions was provided; however this is not a requirement. SAHPRA should then limit the review of the submission to the review of the reference agency assessment report and conduct either an abridged or verification review of certain parts of the technical dossier in support of local requirements. It is recommended that the human pharmacology, quality/chemistry manufacturing and controls and non-clinical data provided in the CTD should only be reviewed in the event of a query. A selective, detailed review of the clinical data provided in the CTD should be performed in order to account for differences in medical practice, national disease patterns, ethnic factors and unmet medical needs. The UMBRA BR Summary Template is recommended for conducting the evaluation of the clinical data and to record the BR decision. It would be highly desirable for assessment reports prepared by SAHPRA during the abridged review process to be peer-reviewed by the scientific committee. More frequent ad hoc consultation of a scientific expert committee should be limited to applications for market authorisation requiring expert review and recommendation.^17^ In terms of publication of a PAR (ZAPAR) and evaluation of QDMPs, the same process as that for the full review is recommended.

###  Regulatory Framework of SAHPRA

**Figure 2:**
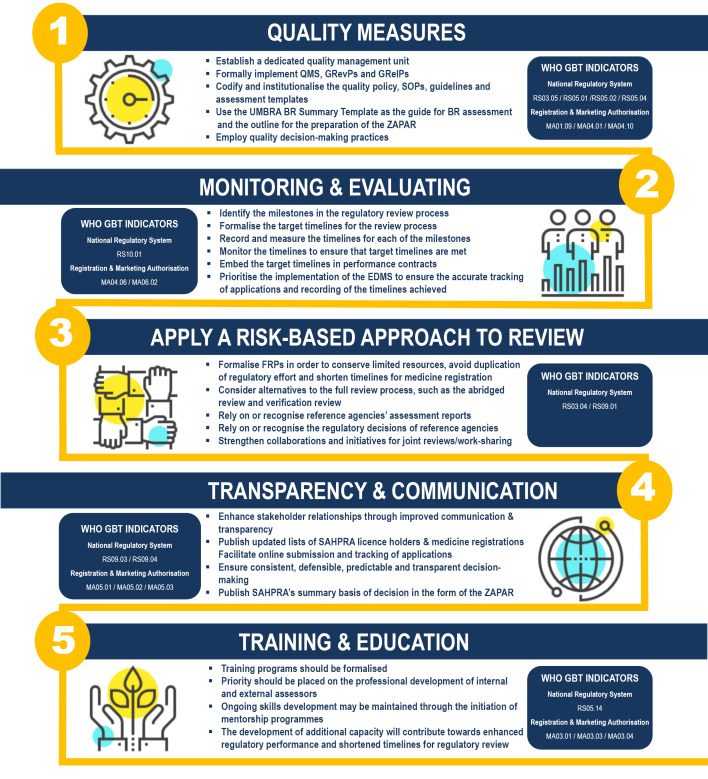
The Proposed Improved Regulatory Review Model for the SAHPRA. Abbreviations: BR, benefit-risk; EDMS, electronic document management system; FRP, facilitated regulatory pathway; GBT, global benchmarking tool; GRP, good regulatory practice; GRevP, good review practice; GRelP; good reliance practice; MA, marketing authorisation; RS, regulatory system; SAHPRA, South African Health Products Regulatory Authority; SOPs, standard operating procedures; QMS, quality management system; UMBRA, Universal Methodology for Benefit-Risk Assessment; WHO, World Health Organization; ZAPAR, South African Public Assessment Report.

 The results have identified inefficiencies in the regulatory framework of SAHPRA and the opportunities for improvement in its regulatory performance.^7,8,10,17-19^ These include: quality measures; monitoring and evaluating review times; a risk-based approach to the evaluation of medicines; transparency and communication; and training and education (Figure 2).

 The WHO GBT sub-indicator MA01.09 specifies that guidelines on the quality, nonclinical/safety and clinical aspects should be established and implemented and should specify the requirements for registration/granting of market authorisation.^23^ The WHO GBT sub-indicator MA04.01 states that documented procedures/tools should be implemented for the assessment of different parts of the application and for the assessment of specific requirements of specific classes of medical products (quality, safety and efficacy).^23^ Both of these sub-indicators endorse the recommendation to formalise the use of the UMBRA BR summary template as a guide for BR assessment and an outline for the preparation of the ZAPAR. In addition, SAHPRA should consider the implementation of QDMPs to support transparent, consistent, predictable and evidence-based regulatory decisions as described in the requirements for sub-indicator MA04.10. The objective of this sub-indicator MA04.10 is to ensure that regulatory decisions are adequately documented and to ensure consistency throughout the review process in terms of requirements and criteria for registration.^23^

####  Quality Measures

 While the MCC had only developed a QMS relating to the activities of the MCC Inspectorate, SAHPRA intends to formalise the establishment of a Quality Management Unit and develop a QMS for the Agency as a whole. However, GRevPs and GRelPs have not been formally implemented; standard operating procedures (SOPs) and templates for the implementation of an abridged review process have not been developed; QDMPs have not been formalised and codified into practice. Although SAHPRA considers BR decision-making through its expert committees, a formalised process documenting BR decisions made by SAHPRA has not been developed or implemented and SAHPRA does not publish assessment reports for NASs.

**Figure 3:**
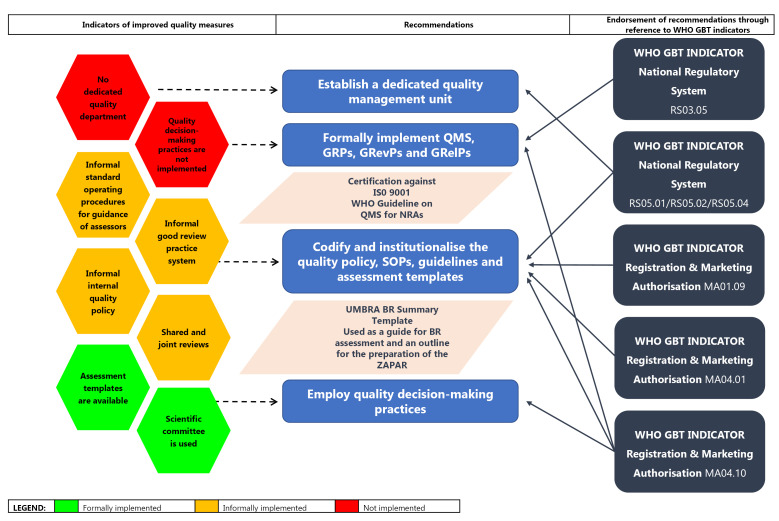
Improved Quality Measures. Abbreviations: BR, benefit-risk; GBT, global benchmarking tool; GRP, good regulatory practice; GRevP, good review practice; GRelP, good reliance practice; ISO, International Standardization Organization; NRA, national regulatory authority; SOP, standard operating procedure; QMS, quality management system; UMBRA, Universal Methodology for Benefit-Risk Assessment; WHO, World Health Organization; ZAPAR, South African Public Assessment Report.

 A dedicated quality management unit should be established and a QMS should be formally implemented. A quality policy, SOPs, guidelines and assessment templates should be codified and institutionalised into practice (Figure 3). These recommendations are endorsed by the WHO GBT sub-indicator RS05.01, which states that top management intervention is required to demonstrate commitment and leadership to develop and implement a QMS; sub-indicator RS05.02, which requires the quality policy, objectives, scope and action plans for the establishment of the QMS to be in place and to be communicated to all levels; and sub-indicator RS05.04, which requires the assignment of enough competent staff to develop, implement and maintain the QMS.^24^ It is recommended that SAHPRA consider following the WHO Guideline on the implementation of QMSs for NRAs^25^ that was developed based on the principles of the ISO Standard 9001:2015 for QMSs. GRPs, GRevPs and GRelPs should also be formally implemented and maintained in order to build quality into the review process. This recommendation is supported by the WHO GBT sub-indicator RS03.05, which requires the NRA to promote GRPs and to ensure that the principles of GRP are applied to the regulation of medicines^24^ and the sub-indicator MA04.10, which requires the formal implementation of GRevPs.^23^

####  Monitoring and Evaluating Review Times

**Figure 4:**
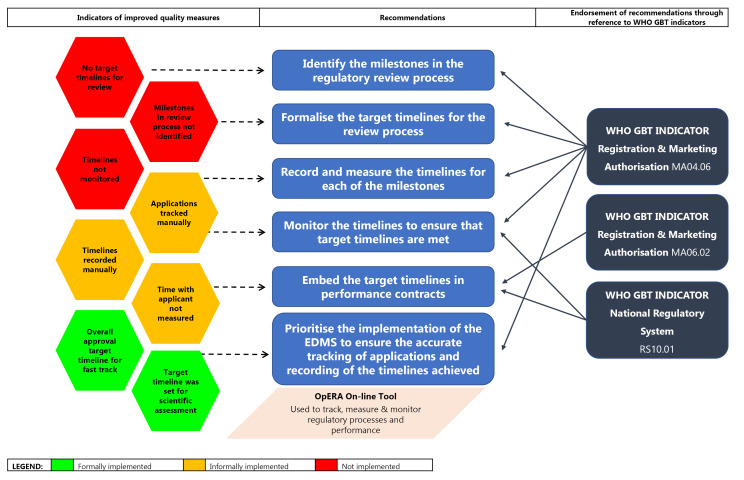
Improved Monitoring and Evaluating of Performance Metrics. Abbreviations: GBT, global benchmarking tool; OpERA, optimising efficiencies in regulatory agencies; WHO, World Health Organization.

 Target timelines and the milestones within the regulatory review process have not yet been identified and formalised. Whist, SAHPRA has identified timelines for overall approval, these are recorded manually and are not monitored routinely. Applications for NASs are also tracked manually. It is, therefore, of paramount importance for SAHPRA to consider identifying the milestones in the regulatory review process and to formalise target timelines for individual milestones as well as the entire review process. The timelines for each of these milestones should be recorded routinely and accurately measured (Figure 4). The data collected should be monitored regularly (quarterly) in order to ensure that target timelines for the review process are continuously met and improved. Thus, the introduction of an EDMS becomes a priority in ensuring the accurate tracking of applications through the milestones of the review process and to provide for the automated and assured collection of the timelines achieved throughout the review process. These recommendations are endorsed by the WHO GBT sub-indicator MA04.06, which requires the establishment of timelines for the assessment of applications and an internal tracking system to follow the targeted timeframes.^23^ Performance contracts should be put in place to ensure that personnel and external assessors responsible for the timely review of medicines are held accountable for achieving the target timelines. This is supported by the WHO GBT sub-indicator MA06, which requires the use of a mechanism to monitor regulatory performance and output^23^; sub-indicator MA06.02, which requires the establishment and implementation of performance indicators for registration and/or market authorisation activities;^23^ and the sub-indicator RS10.01, which requires the monitoring, supervision and review of NRA and affiliated institution performance using key performance indicators.^24^

####  Risk-Based Approach to the Evaluation of Medicines

 SAHPRA, to date, has not publicly formalised the implementation of a risk-based approach to the review of NASs. Policies, SOPs and templates for FRPs have not been developed while target timelines and milestones have not currently been identified and formalised.

**Figure 5:**
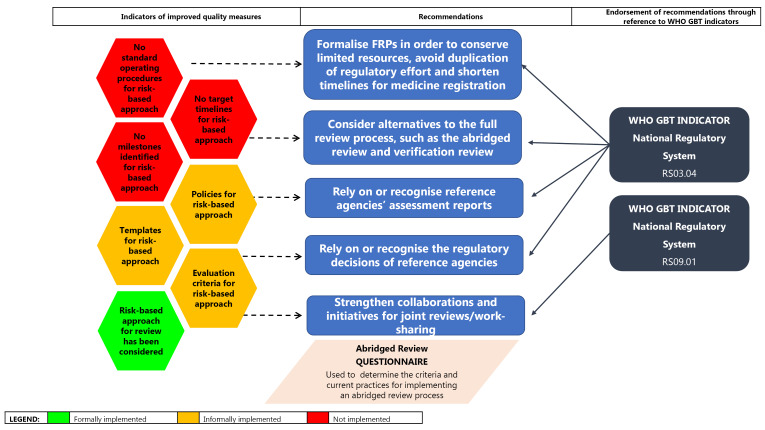
Application of a Risk-Based Approach to Medicine Review. Abbreviations: FRP, facilitated regulatory pathway; GBT, global benchmarking tool; WHO, World Health Organization

 It is critically important that SAHPRA, as a newly established NRA, consider applying a risk-based approach to the regulatory review of medicines whereby the allocation of resources is commensurate with product risk. FRPs should be formalised in an effort to conserve limited resources, to avoid duplication of regulatory effort and shorten timelines for medicine registration. SAHPRA has considered alternatives to the full review process, such as the abridged and verification review as well as recognition and has also considered placing reliance on the assessment reports of the regulatory decisions of reference agencies. Initiatives for joint reviews or work sharing should be further developed to support continued enhancement of regional initiatives such as Zazibona and continental and international collaborations (Figure 5). These recommendations are endorsed by the WHO GBT sub-indicator RS03.04, which supports the formalisation of reliance on the decisions of other mature NRAs through documented policy, procedures and/or mechanisms and the sub-indicator RS09.01, which encourages NRAs to participate in a regional and/or global network in order to promote convergence and harmonisation efforts.^24^

####  Transparency and Communication

**Figure 6:**
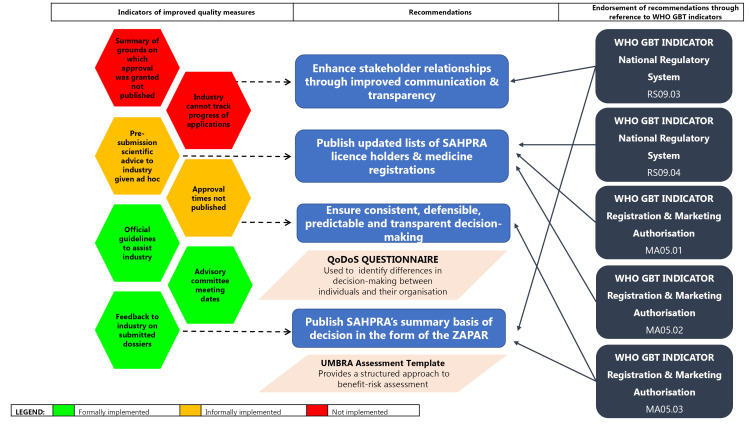
Improved Transparency and Communication. Abbreviations: GBT, global benchmarking tool; QoDoS, quality of decision-making orientation scheme; SAHPRA, South African Health Products Regulatory Authority; UMBRA, Universal Methodology for Benefit-Risk Assessment; WHO, World Health Organization; ZAPAR, South African Public Assessment Report.

 SAHPRA has not implemented an online system for the submission of applications for registration and the tracking thereof and does not publish PARs nor negative regulatory decisions. These findings indicate that SAHPRA should also consider adopting improved communication strategies and increased transparency, which would in turn enhance stakeholder relationships (Figure 6). In addition, the SAHPRA website should be supplemented with the publication of updated lists of licence holders and medicine registrations. Furthermore, this would need to be underpinned by the development, implementation and maintenance of appropriate information and communication technology solutions to facilitate the online submission of applications supported by systems that allow the industry to track the progress of their applications.

 Our findings and the associated recommendations described above are supported by the WHO GBT indicator MA05, which highlights the need for the NRA to ensure that mechanisms exist to promote transparency, accountability and communication. These recommendations are further endorsed by the sub-indicator MA05.01, which requires the NRA to ensure the availability of a website or other official publication that is regularly updated^23^; sub-indicator MA05.02, which requires the publication of an updated list of all medicines granted market authorisation;^23^ and the sub-indicator RS09.04, which requires the publication of information on marketed medical products, authorised companies and licensed facilities.^24^ The Agency could ensure consistent, evidence-based predictable and transparent decision-making through considering the adoption and application of the UMBRA BR Summary Template for BR assessment and the publication of SAHPRA summary basis of decisions in the form of the ZAPAR. This recommendation is endorsed by the sub-indicator MA05.03, which requires the publication of summary technical evaluation reports for approved applications of marketing authorisation in the public domain^24^ and the sub-indicator RS09.03, which requires the publication of the NRA decisions related to regulatory activities in the public domain.^24^ The placement of the ZAPAR in the public domain will also support and strengthen the position of SAHPRA as an NRA whose regulatory decisions may be relied on or recognised by other similar NRAs in the emerging economies.

####  Training and Education

**Figure 7:**
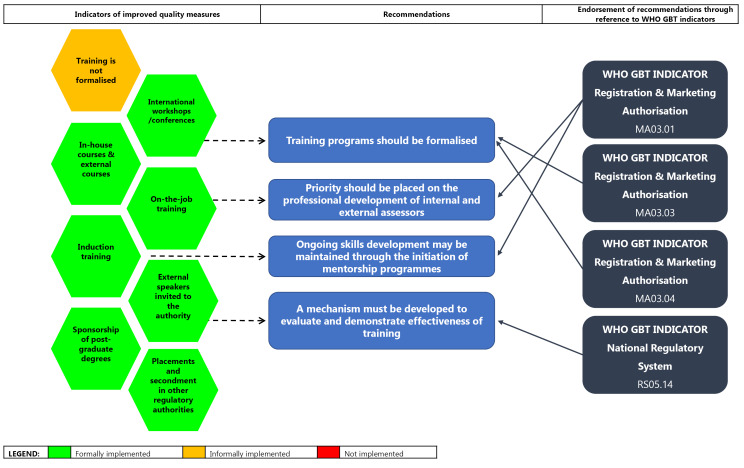
Improved Training and Education. Abbreviations: GBT, global benchmarking tool; WHO, World Health Organization.

 SAHPRA has not as yet formally implemented training and mentorship programmes, apart from ad hoc technical training and orientation programmes offered to staff. Training programmes should be formalised and priority should be placed on the professional development of both internal and external assessors (Figure 7) as well as administrative personnel. Ongoing skills development may be maintained through the initiation of mentorship programmes. These recommendations are endorsed by the requirements of the sub-indicators of the WHO GBT such as: MA03.01, which states that sufficient competent staff (education training skills and experience) should be assigned to perform marketing authorisation; MA03.03, which requires the development, implementation and annual updating of the training plan; MA03.04, which describes the requirement of performing and maintaining records of staff training activities^23^; and RS05.14, which requires the establishment of a mechanism to evaluate and demonstrate the effectiveness of training activities.^24^ Ensuring the development of additional capacity through training and education will contribute towards enhanced regulatory performance, shortened timelines for regulatory review and retention of skilled staff.

## Discussion

 The historical context and the evolution of the legislation supporting the transition of the MCC to the newly established SAHPRA has been reviewed.^10^ The challenges and opportunities for the regulatory transformation of SAHPRA and achieving improved regulatory responsiveness and performance have been identified. For the first time, studies were undertaken using well-defined methods and techniques to evaluate the regulatory review process as it was applied by the MCC,^7,8^compared the MCC review process to that of other similar-sized NRAs,^17^ analysed the inherent differences in the operational model of the MCC compared to SAHPRA^10^ and made key recommendations for the improvement of the regulatory review process as it may be applied by SAHPRA. The level of implementation of quality measures, good regulatory and review practices, decision-making practices and continuous improvement initiatives by the South African NRA has been assessed^7^ and an evaluation of the guidelines and templates newly developed and initiated by SAHPRA, addressing the historical limitations in the application of FRPs, has been performed for the first time. As a result, recommendations for an improved model for the regulatory review of medicines have been proposed.

 These studies^7,8,10,17-19^ have been valuable in providing a baseline against which the results of the recommended improvements to the reformed regulatory review process under SAHPRA may be quantitatively evaluated and presented. Following the implementation of the SAHPRA re-engineered processes it would be useful to reflect on its revised organisational structure, regulatory review process and regulatory performance; evaluate its performance metrics and overall median approval times for NASs (2019-2020) and compare its new registration process and regulatory review model against other similar-sized NRAs.

 Provided that the recommendation, to identify and routinely monitor and evaluate the milestones in the regulatory review process, is implemented, it would be useful to analyse the timelines achieved between these milestones in order to accurately determine the time taken by SAHPRA to review an application for the registration of NASs and the time taken by the applicant to provide the required response/s to SAHPRA. It is evident from recent studies that SAHPRA needs an action plan for an improved regulatory review model in order to decrease the timelines for approval of NASs and accelerate patients’ access to new medicines.^7,8,10,17-19^ To achieve this, it is recommended that SAHPRA makes provision for an online application process supported by an effective EDMS to support the tracking of applications and the monitoring and evaluation of the milestones and timelines within the regulatory review process.

###  Reliance and Work-Sharing

 A number of key recommendations, underpinned by GRPs, GRevPs and GRelPs, have been developed and are considered to be the core elements required to support the proposed improved regulatory review model for SAHPRA. The implementation of these recommendations is crucial in meeting the requirements of several of the sub-indicators within the WHO GBT that contribute towards the regulatory performance of a sustainable and efficient regulatory system. These recommendations are considered to be fundamental for SAHPRA to achieve a maturity level rating of either 3 or 4 and become a WHO-listed NRA. As a WHO-listed NRA, SAHPRA would be in a position to serve as a reference agency to other NRAs within the African region, thus advancing the contribution of SAHPRA in both regional and continental reliance and work-sharing initiatives. The drive for the implementation of collaborative initiatives to support the appropriate allocation of limited resources and to reduce the duplication of regulatory effort has been observed.^5^ SAHPRA has participated in such initiatives, most notably the regional Zazibona collaborative registration process. The application of a common model, such as this one, will promote alignment in regulatory review processes and will support the development of expanded regional models for work sharing and joint assessment. Region-wide application of this model has the potential to establish the African region at a new level of regulatory efficiency as well as accelerating patients’ access to new medicines.

###  Limitations and Future Work

 This study was limited to a review of the South African regulatory environment, however, the model that has been developed through this study provides a road map that could be implemented by other NRAs to achieve enhanced regulatory performance. In the light of the similarities in the challenges faced by NRAs in the emerging economies, it is highly likely that this model could be applied by such NRAs within the African region and beyond.

 It would be valuable to study the regulatory performance and the opportunities for the enhancement of both regional and continental collaborative initiatives in Africa. Future work could include interviewing regulatory agencies that have implemented an abridged review process to determine the criteria and current practices for implementation. This information would provide insight into how FRPs may be used to strengthen the regulatory performance of the Zazibona collaborative initiative or work-sharing/joint reviews in the South African Development Community region or within the African continent.

## Conclusion

 The findings of the studies reported here have led to a series of key action plans for the development of an improved model for regulatory review, a model for BR assessment supported by quality decision-making as well as recommendations for the application of risk stratification strategies, strengthening of reliance networks, reinforcing GRPs and enhancing transparency. It is hoped that the proposed improved model will be considered by SAHPRA and will pave the way towards efficient and transparent, streamlined review processes, coupled with increased consistency, evidence-based decision-making practices, reduced timelines and improved patients’ access to new medicines.

## Acknowledgements

 The OpERA programme is underwritten by the Centre for Innovation in Regulatory Science (CIRS) and is supported by a grant from the Bill and Melinda Gates Foundation.

## Ethical issues

 The study received Ethics approval from the University of Hertfordshire Institutional Ethics Committee (LMS/PGR/UH/03873).

## Competing interests

 Authors declare that they have no competing interests.

## Authors’ contributions

 AK developed the study design, collected and analysed the data, interpreted the results and wrote the first draft of the manuscript. SS: Developed the study design, provided guidance for the data collection and analysis, interpreted the results and reviewed the manuscript. SB provided guidance on the interpretation and relevance of the results, liaised with SAHPRA and reviewed the manuscript. SW developed the study design, liaised with the regulatory authorities for data acquisition, provided guidance for the data collection and analysis, interpreted the results and reviewed the manuscript.

## Authors’ affiliations

^1 ^Department of Clinical and Pharmaceutical Sciences, School of Life and Medical Sciences, University of Hertfordshire, Hatfield, UK. ^2^South African Health Products Regulatory Authority, Pretoria, South Africa. ^3^Faculty of Health Sciences, University of the Witwatersrand, Witwatersrand, South Africa. ^4^Centre for Innovation in Regulatory Science, London, UK.
